# Interior-Point Methods for Estimating Seasonal Parameters in Discrete-Time Infectious Disease Models

**DOI:** 10.1371/journal.pone.0074208

**Published:** 2013-10-22

**Authors:** Daniel P. Word, James K. Young, Derek A. T. Cummings, Sopon Iamsirithaworn, Carl D. Laird

**Affiliations:** 1 Department of Chemical Engineering, Texas A&M University, College Station, Texas, United States of America; 2 Bloomberg School of Public Health, Johns Hopkins University, Baltimore, Maryland, United States of America; 3 Bureau of Epidemiology, Ministry of Public Health, Bangkok, Thailand; Ben-Gurion University, Israel

## Abstract

Infectious diseases remain a significant health concern around the world. Mathematical modeling of these diseases can help us understand their dynamics and develop more effective control strategies. In this work, we show the capabilities of interior-point methods and nonlinear programming (NLP) formulations to efficiently estimate parameters in multiple discrete-time disease models using measles case count data from three cities. These models include multiplicative measurement noise and incorporate seasonality into multiple model parameters. Our results show that nearly identical patterns are estimated even when assuming seasonality in different model parameters, and that these patterns show strong correlation to school term holidays across very different social settings and holiday schedules. We show that interior-point methods provide a fast and flexible approach to parameterizing models that can be an alternative to more computationally intensive methods.

## Introduction

Infectious diseases continue to be a significant public health concern, especially in developing countries where inadequate resources, social influences, and environmental factors may prevent effective, sustained results from public health initiatives [1], [2]. From a public health perspective, it is clear that reliable models can greatly aid in the decision making process. For example, quantitative long-term dynamic models could be used to determine optimal allocation of limited resources, assess the effectiveness of current public health practices, or even predict outbreak risk. From a scientific perspective, the identification of a reliable mechanistic model can improve our understanding of the important factors affecting infectious disease dynamics.

Both of these goals require estimation of parameters in infectious disease models from empirical data. Childhood diseases like measles and chickenpox, for which long-term case count data is available, provide an appropriate test bed for developing models and estimation procedures. Probably the most highly studied dataset for measles has been made available electronically by Grenfell [3] (URL: http://www.zoo.cam.ac.uk/zoostaff/grenfell/measles.htm). This data set contains yearly reported birth records in addition to biweekly reported measles case counts and has several favorable properties, including a high reporting fraction and temporal resolution. Unfortunately, these favourable characteristics are not typical among other datasets, including one studied in this paper.

For most long-term studies, the only available data are disease case counts (incidence) aggregated over time periods that are longer than the serial interval for the disease (typically monthly or even quarterly). Little information is known about the number and dynamics of susceptibles within the population, a critical determinant of disease dynamics; therefore this state variable must be estimated along with the unknown parameters. Furthermore, incidence is almost always under-reported since data is typically collected passively, health care providers may neglect to report all cases, and some cases may not be accurately diagnosed. The reporting fraction is difficult to quantify, but can be significantly lower than unity, and must be considered in the estimation procedure. Changes in public health policies and administration, as well as changes that affect the population at risk, and changing geographic boundaries such as city expansion, can result in reporting inconsistencies over the full time horizon. While each of these difficulties may not be present in all epidemiological datasets, they are representative of those encountered in many historical, passively-reported datasets on the incidence of childhood diseases, and they result in significant challenges for effective parameter estimation.

In addition to the difficulties inherent in the available data, estimation is further complicated by the structure of disease models themselves. There are two fundamental classes of mechanistic models used for the spread of infectious disease. Individual or agent-based modeling approaches have been used extensively, however, the large parameter space of these models often overwhelms the data available to specify those parameters. The classic framework of compartment models (e.g. the Susceptible-Infected-Recovered (SIR)) have fewer parameters and can be described by sets of differential or discrete-time equations, allowing for efficient, derivative-based estimation from historical case data. Within this framework, model structures can vary dramatically depending upon the selection of incidence and recovery functions, the discretization strategy selected, and the consideration of age or spatial dynamics. Furthermore, when performing parameter estimation, several measures of fit can be proposed. Therefore, we present an estimation formulation and solution procedure that is flexible enough to accommodate the limitations in the available data and the challenges associated with complex nonlinear models. In addition, this approach is computationally efficient and allows for exploration of different model structures along with the promise of tackling larger systems.

Advanced NLP packages provide an excellent framework for efficient estimation of infectious disease models from long-term case data. Modern mathematical programming languages (e.g., AMPL [4], GAMS [5], Pyomo [6]) provide efficient computation of derivative information through automatic differentiation in a flexible framework for exploration of different model structures. In this paper, we present an approach for estimating parameters in a discrete-time SIR model. The model equations are included as equality constraints in the NLP problem. In this approach, the values of the system states are converged simultaneously with the model parameters. This technique has the potential to be very efficient since the forward problem is converged only once along with the estimation problem. We demonstrate this approach by formulating three models with seasonality included in different model parameters. The first model estimates seasonality in the transmission parameter, the second seasonality in an exponential parameter on the incidence, and the third seasonality in the introduction of new susceptibles into the population (i.e. from births). Our results show that the estimated seasonality is strongly correlated to school holidays across multiple settings regardless of how seasonality is included in the problem formulation. Solution times demonstrate the efficiency of our approach, with the longest run-time for any of our estimations requiring less than 6 seconds, even when considering over 20 years of historical data. The efficiency and flexibility of this estimation framework make it suitable for investigation of new model structures and increasingly larger problems.

In the section titled “Background”, we discuss the relevant literature and, in particular, the seasonality of measles transmission and the formulation of the time-series SIR model. In “Methods”, we present the NLP problem formulations used for parameter estimation and an overview of the interior-point strategy for solving these large-scale NLPs. The section titled “Data” describes the data and data preparation used for our estimations. The section titled “Results and Discussion” shows our estimation results for a seasonal transmission parameter performed using real measles case count data from London, New York City, and Bangkok. We also present results from problem formulations that contain seasonality in model parameters other than the traditional transmission parameter. The section titled “Conclusions” presents the significance of our results, and we mention topics for future research in “Future Work”.

### Background

The basis for the formulation of traditional infectious disease models is the compartmental framework where the population is divided into various compartments based on their status with respect to the disease [1], [7]–[9]. In the basic SIR model, the population is assumed to be well-mixed, and individuals are classified as being susceptible to the disease (S), infected with the disease (I), or recovered from the disease and currently immune (R). Mathematical models based on the compartmental framework can be formulated in both continuous-time (resulting in coupled differential equations) and discrete-time (giving rise to a large set of algebraic or transcendental equations). For a discrete-time model, 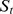, 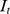, and 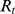 are the current number of susceptible, infected, and recovered individuals at each time interval 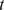. The infection process that defines the number of new cases in a given time interval is described through the incidence function, which usually depends on the present value of the state variables as well as model parameters 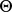, which may themselves depend on time.

The classic incidence function for the number of new cases at interval 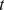 is typically defined by 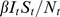. Here, 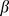 (known as the transmission parameter) is proportional to the number of adequate contacts for the spread of infections, and 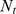 is the total population at time 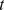, which is typically known from census data. Different models have been proposed for the recovery function, including a constant time delay (individuals stay infected for a fixed period of time) and a fixed recovery rate (resulting in an assumption of exponentially distributed recovery times). In a structural sense, this is one of the most basic models of infectious disease dynamics, and more complex compartment structures have been studied [1], [7], [10]. However, significant flexibility in this basic model is still possible through various definitions of the incidence and recovery functions. In particular, by allowing seasonal model parameters, discrete-time models of this basic structure have a tremendous capacity to fit real world case data [3], [11], [12].

Based on work by Soper [13], Fine and Clarkson consider an incidence function where the transmission parameter is allowed to vary with time [14]. They estimate values of this temporally varying transmission parameter using measles incidence data collected in England and Wales from 1950–1966. Over this time period, the incidence follows a biennial pattern of alternating major and minor epidemics. Remarkably, however, their estimate of the time-varying transmission parameter has a similar pattern and magnitude in both minor and major epidemic years. Furthermore, this pattern is loosely correlated with school holidays. This strongly supports the assertion of a relatively consistent, underlying seasonal transmission effect related to school terms. They conclude that the observed biennial pattern is a result of the dynamics of susceptible individuals in the population as driven by births and the infection process.

Semi-mechanistic approaches are also used to describe the infection process. Ellner et al. couple the mechanistic compartment balances with a phenomenological model (empirically estimated) to describe the incidence function [15]. Using the measles dataset from England and Wales, they estimate a general form for the incidence relationship using both feed forward neural networks and semi-nonparametric models. Probing the input-output behavior of their estimated incidence relationship, they suggest the presence of an underlying seasonal effect and that the incidence function should be nonlinear in 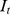. Cauchemez and Ferguson confirm that assuming nonlinearity in 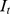 is necessary to not miss key features of epidemics [16]. Lui et al. conduct a thorough analysis of the equilibrium behavior and stability properties of various continuous-time compartment models with nonlinear incidence rates of the form 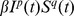 [17]. They conclude that, while values of 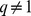 have no “major effects”, altering the value of 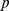 from unity can have a significant effect on qualitative, long-term behavior. Word et al. do not assume nonlinearity in 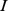 and find reliable estimates for seasonal transmission parameters using a continuous-time model [18].

There is significant work being done to investigate the homogeneity, or lack thereof, in mixing within populations. Keeling and Eames explore the implementation of various techniques from network theory into epidemiology theory to provide a more accurate estimation of mixing networks than the typical random-mixing assumption [19]. In another paper, Keeling specifically examines using metapopulation models to better understand mixing dynamics [20].

The results of Fine and Clarkson and others provide strong evidence of an underlying seasonal mechanism that is linked to school terms [14], although Gomes et al. did not reach that conclusion for Portugal [21]. However, the Fine and Clarkson model [14] has been criticized [22], since its long term behavior does not exhibit the observed two-year periodicity, but rather a periodicity close to three years. It is with this backdrop that Finkenstädt and Grenfell introduce the time-series SIR model that can adequately describe the periodicity of the data by using a seasonally varying transmission parameter [11].

We extend the ideas of Finkenstädt and Grenfell [11] and present a large-scale, NLP approach for efficient estimation of time-series SIR models using existing case data. We make use of the time-series SIR model because of its demonstrated ability to reliably represent measles time-series data and the lack of an assumed functional relationship restricting the shape of the seasonal transmission profile. However, unlike in [11], our approach is not one-step-ahead, and we do not require the susceptible dynamics and the time-varying reporting fraction as inputs, but instead estimate them simultaneously with the unknown model parameters. The simultaneous estimation of the reporting fraction has been performed previously with a continuous-time SEIR (Susceptible-Exposed-Infected-Recovered) model, where a generalized profiling estimation approach was used that also estimated the susceptible dynamics and the reporting fraction along with the model parameters [23]. Here, we use an interior-point method to estimate seasonal parameters for discrete-time models and investigate seasonality in multiple model parameters.

Our estimation problem is formulated as a large-scale NLP problem. The discrete-time model is written over the entire time horizon of the selected data and included in the formulation as constraints. While this approach produces a large-scale NLP problem, it can be solved efficiently using modern NLP solvers. Advancements in NLP algorithms, including the introduction of large-scale nonlinear interior-point methods [24]–[28] allow efficient solution of increasingly large problems. In addition, this approach is very flexible, allowing easy formulation of new model structures. While previous work has shown benefits of using continuous-time models rather than discrete-time models [18], [29], discrete-time models are still commonly used because of their simplicity and their ability to adequately describe the observed data. This fact drives the development of the efficient solution approach presented here.

The effectiveness of this overall approach is demonstrated with data from communities at a time where measles was endemic and monthly case counts remained above zero for decades. Parameters are estimated using available measles data from the UK for 1944–1964 [3] and New York City for 1944–1963 [30], as well as pre-vaccination measles data collected in Thailand from 1975–1986 [31]. The school term schedule in Thailand differs significantly from the schedule in the other two locations, making it an excellent complimentary dataset for comparing the seasonality of model parameters with school patterns.

## Methods

In this section, the base problem formulation is introduced, along with a description of the sparse interior-point method used to solve the large-scale nonlinear problem. We describe three estimation formulations that incorporate seasonality into different model parameters. The first formulation estimates a seasonally varying transmission parameter 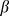 (labeled SVTP). The second formulation estimates a seasonally varying exponential parameter 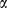 (labeled SVEP). The third formulation estimates seasonality in the introduction of susceptibles into the population (labeled SVIS).

### Estimation Problem Formulations

The deterministic skeleton of the TSIR (Time-series SIR) model used in this analysis is given by,
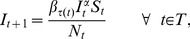
(1)

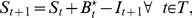
(2)where 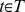 refers to the set of discrete time periods, 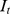 is the number of new cases in time period 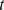, 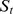 is the current number of susceptible individuals, and 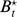 corresponds to the number of yearly births divided by the number of discrete-time intervals per year. Equation 1 describes the infection process and Equation 2 is the susceptible balance. Note that the discrete time interval is assumed to be the same as the generation time of disease and, as such, removes the distinction between incidence and prevalence. Full recovery is assumed from one time interval to the next. The transmission parameter 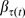 is seasonal and restricted to be the same from year to year (i.e. 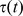 is a mapping from the overall time interval 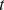 to an index from the beginning of the current year only). The exponent 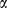 is a parameter that allows a nonlinear dependence on 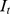 in the incidence function [2], [3], [11], [12], [17]. In this model, population 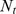 and births 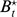 are known inputs.

The goal is to estimate the unknown parameters 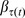 and 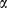 along with the unobserved state 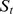 using reported incidence. However, the cases are almost always under-reported. Therefore, the true incidence 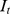 is related to the reported incidence 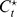 by an unknown, potentially time-varying, reporting fraction 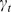,
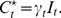
(3)


In the absence of additional information or further restriction of the time-varying reporting fraction, it is clear that unique estimation is not possible. Any value for 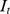 can be matched exactly to the reported incidence 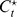 by setting 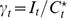. In our work, we will assume that the reporting fraction varies linearly over the entire time horizon, although this framework supports general restrictions on its functional form. Additionally, we assume multiplicative measurement noise in the reported cases since the variance of the noise appears to increase with the number of reported cases [11] (i.e., 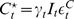 where 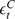 is an unknown error term).

To improve the scaling and convergence properties of the nonlinear estimation formulation, an exact log transformation is performed on the incidence expression and the reporting fraction correction expression. This gives the formulation of our discrete-time deterministic model with seasonally varying transmission parameter (SVTP), 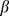, shown in Problem 4.
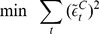
(4a)s.t.
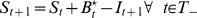
(4b)

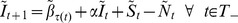
(4c)

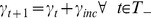
(4d)

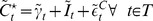
(4e)

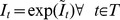
(4f)

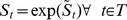
(4g)

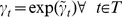
(4h)

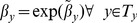
(4i)

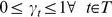
(4j)

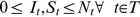
(4k)Here, 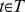 refers to the discrete time interval in the entire time horizon 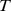, set 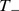 is identical to set 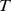 except that it is missing the last element of 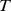, and 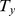 is the set of time intervals within a single year. 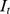 is the number of new cases at time 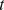, 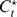 is the number of reported cases at time 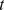, 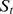 is the number of susceptible individuals at time 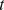, 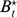 still corresponds to the number of yearly births divided by the number of discrete-time intervals per year, and 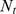 is the population at time 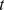. The variable 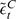 is the log transformation of the multiplicative error in the number of reported cases, 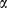 is an exponential parameter on the incidence, and 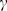 is the incidence reporting fraction that is assumed to vary linearly by some increment 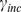 between each time interval. The seasonal transmission parameter 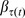 is restricted to be the same from year to year (i.e. 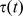 is a mapping from the overall time interval 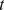 to an index 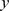 from the beginning of the current year only). The ∼ symbol denotes log-transforms such that 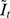, 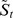, 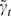, 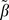, and 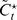 are the log-transformations of 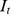, 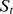, 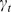, 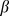, and 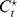 respectively. Note that this model uses exact log transformations and not linear approximations.

This formulation has several advantages over previously existing approaches for discrete-time models. This approach simultaneously estimates the susceptible dynamics and the reporting fraction along with the disease parameters, and provides an estimate of the susceptible count profiles in time. In addition, this formulation can easily account for missing data by removing terms from the objective function for periods where no data is available, and given the flexible nature of the framework, a variety of measures of fit could be used as the objective function.

The estimation formulation (SVTP) above assumed seasonality in the transmission parameter 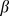, however, it is reasonable to postulate models with seasonality in other model parameters. In an effort to better understand potential drivers of observed infectious disease dynamics, this paper also presents estimation results for formulations that include unknown seasonality in the exponential parameter 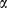, and in the birth rate. In particular, we wish to know if alternate models provide improved fit to the data, and if the estimated seasonal patterns are the same for different parameters (e.g., are they still correlated with school holiday schedules). The first alternative model formulation includes a time-invariant transmission parameter but seasonal exponential parameter 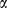. This formulation is identified as SVEP and is identical to that shown in Equation 0 except that the incidence balance (4c) is now
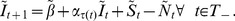
(5)Here, 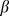 is no longer seasonal, but 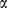 is defined to be seasonal where 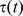 is a mapping from the overall time interval 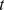 to an index from the beginning of the current year only.

In the third formulation, we investigate estimation of seasonality in the birth rate. Our available birth data includes the yearly number of births only, and in previous formulations, we have assumed that births occur uniformly throughout the year. However, time-varying birth rates may contribute to susceptible dynamics in a non-uniform way throughout the year due to the newborn children effectively entering the pool of individuals at risk of infection at particular times of year. Therefore, we developed a formulation that estimates unknown seasonality in the birth data from case count data to see if seasonality observed in infection data can be captured by seasonally varying births. It is important to note that the estimated seasonal pattern may not be directly related to seasonality in the births themselves, but rather when those births provide the most impact on the observed dynamics (e.g. school entry).

The model formulation assuming seasonality in births, identified as SVIS, incorporates several differences from the previous formulations. Rather than assuming a uniform addition of births into the susceptible population throughout the year, this formulation includes a weighting factor 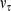 that allows for seasonal variation in births, keeping 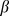 and 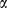 time-invariant. This model formulation differs from that in Equation 4 by replacing the susceptible balance (4b) with (6), the incidence balance (4c) with (7), and adding one new constraint (18),
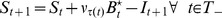
(6)

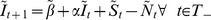
(7)

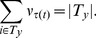
(8)Here, 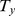 refers to the set of discrete time within a single year, 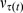 is a seasonally varying weight on births, and 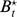 correspond to the number of yearly births divided by the number of discrete-time intervals per year. Equation 8 ensures that the number of new susceptibles introduced into the population every year is equal to the number of reported births for each year, and 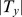 is the cardinality of 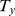 (i.e., the number of discrete time intervals per year). However, this constraint allows these new susceptibles to be added in a seasonally varying manner.

In this paper, we estimate parameters using the large-scale, full-space interior-point method, Ipopt [24], [32]. The Ipopt algorithm implements a primal-dual log-barrier interior-point approach for handling large-scale nonlinear (and non-convex) programming problems that may have many variable bounds. The Ipopt algorithm makes use of full first and second order derivative information for the constraints and the objective. In this research, the modeling language AMPL [4] was used to describe the problem formulation. AMPL provides efficient numerical values of the analytical derivatives through automatic differentiation. The original implementation of the algorithm was developed in Fortran by Andreas Wächter and Lorenz T. Biegler. For full details of the algorithm please see the literature [24], [33].

### Data

Four different data sets were considered in this work. Simulated data from an SIR model was used to validate the estimation procedure. Case data from London was used to compare our estimation results with existing literature values. The London data set has been heavily studied and is used in this work to demonstrate the agreement of our approach with existing literature. Data from two cities, New York City (NYC) and Bangkok, are investigated in this study using all 3 estimation formulations. The NYC and Bangkok data sets are used due to their very different school term holidays, allowing us to show the correlation between school terms and seasonal transmission. NYC has a long summer school holiday lasting from the end of June until mid September, while Bangkok has two long school holidays: one from the beginning of March until the middle of May and one the entire month of October.

The data from London reports biweekly measles cases and yearly birth rate data for the years 1944–1963 [2]. A constant population was assumed for this data set. The data from New York City (NYC) contained yearly reported population and birth rate data, and monthly reported measles case counts for the years 1944–1963 [30]. The Bangkok data contained yearly reported birth rate data [31], but the population was only reported every decade. Linear interpolation was used to approximate the yearly populations across the time horizon studied. The measles case counts were reported monthly for the years 1975–1986. In all of these estimations, the population is assumed to vary linearly throughout each year, and the birth rate is assumed to be uniform throughout each year.

In our estimation approach, we assume that the under-reporting of incidence varies linearly in time, and we estimate a reporting fraction along with other model parameters. An additional challenge in the Bangkok data is the absence of case count data for the year 1979. To account for this, the formulation is modified to exclude these points from the objective function, while still including them in the simulated dynamics.

To estimate the discrete-time model, data must be available on the same time interval as the model discretization. The London data is available in a biweekly form that is consistent with the model discretization. However, the NYC and Bangkok data were reported monthly, so the data must be converted into a biweekly form. To resample the data, the monthly case counts are first converted to cumulative case counts. This cumulative data is interpolated at biweeks with a piecewise cubic Hermite interpolating polynomial (using the pchip method in MATLAB) to ensure no overshoot. The number of new cases in the biweekly intervals was then estimated by taking the difference between the biweekly interpolated data points.

## Results and Discussion

In this section, we present estimation results from the three different formulations. All models assume multiplicative noise in the measurements but differ in how seasonality is included in the model. First, we validate our estimation procedure on simulated data using a model incorporating the common assumption of seasonality in the transmission parameter 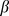. Additionally, we perform estimation on real measles case count data from London and compare our results with other literature studies. After validating our estimation procedure, we present estimates for seasonal profiles using measles data from New York City and Bangkok. These two data sets are used for estimates with all three model formulations.

Confidence intervals and regions are found using the log-likelihood method presented in Rooney and Biegler [34]. Confidence intervals for all estimated parameters are constructed by fixing one parameter and allowing optimization over the remaining parameters. Confidence regions are created by fixing the 2 parameters being compared and optimizing all other variables. These confidence regions show the relationship between the exponential parameter (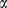), the mean of the transmission parameters (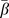), and the mean susceptible fraction (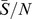).

### Model and Procedure Validation

We first test the SVTP (4) estimation formulation using known parameter values. We perform 10,000 simulations with an SIR model using MATLAB. Our simulations use a constant population of 10,002,000, a birth rate of 2.5% of the population per year, and a reporting fraction of 0.5. To generate 20 years of case data, the deterministic model is simulated for 100 years to achieve a cyclic steady state, and the final values from this simulation are used as the initial values for the 20 year simulation. The simulations are performed with the same model as that used for the estimation. Multiplicative measurement noise is drawn from a log-normal distribution with mean 1 and a standard deviation of 0.1 and applied to the reported cases.

**Figure 1:**
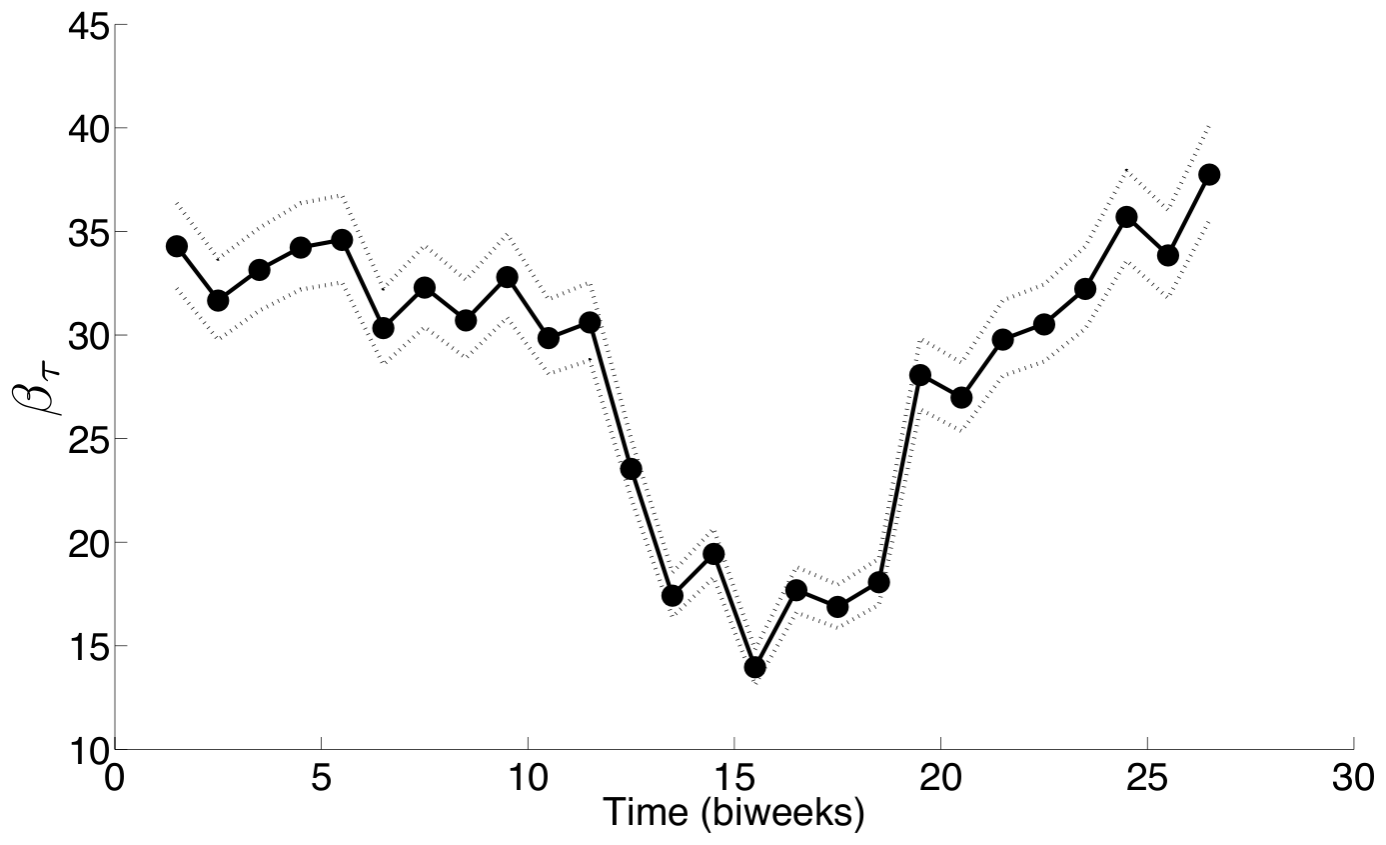
The true values of 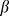 used in the SIR simulation study (circles). The mean of the estimates from the simulation study (solid line). The 2.5th and 97.5th quantiles of the estimates from the simulation study (dashed lines).

**Figure 2:**
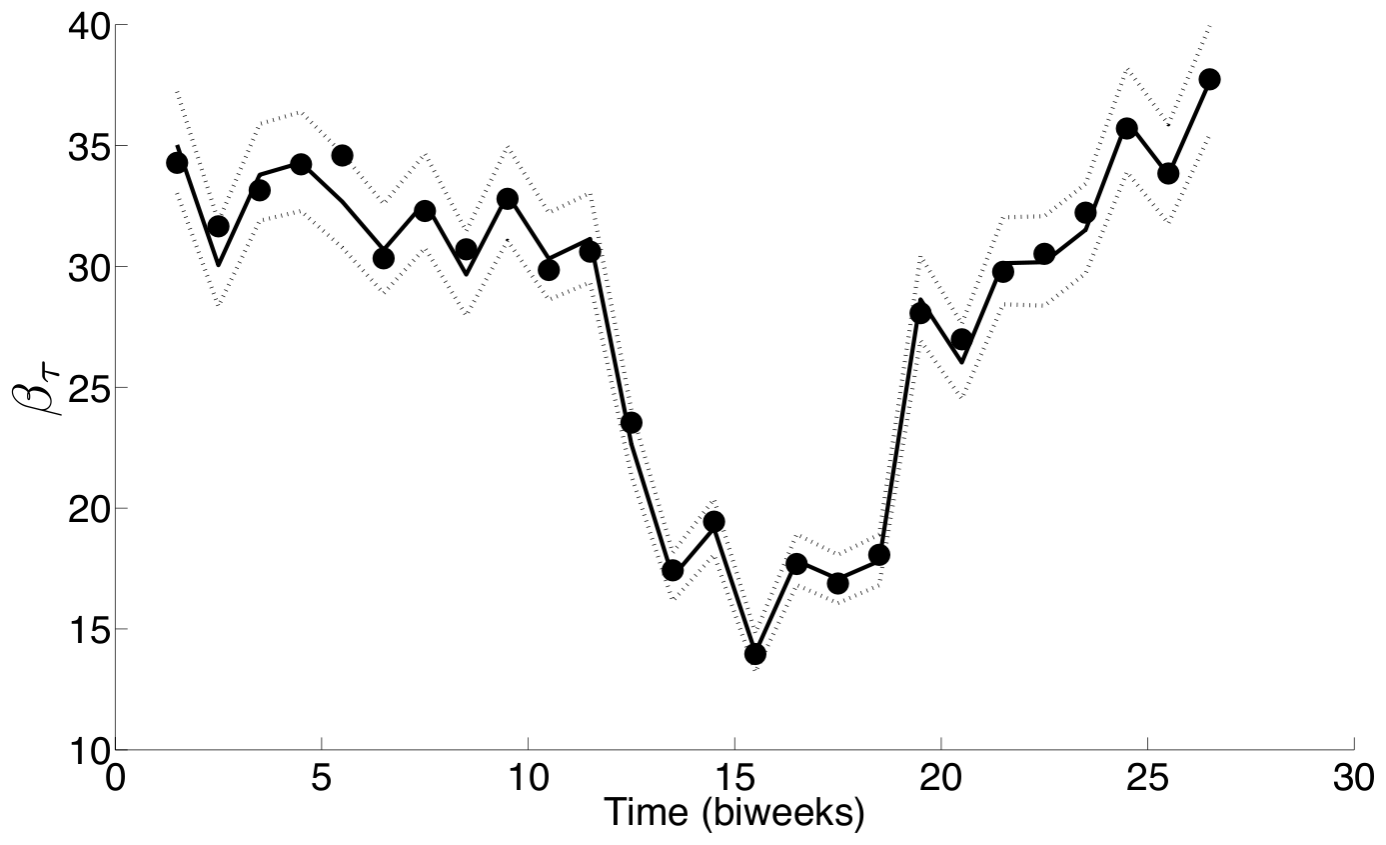
The estimated transmission profile 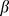 (solid line) for a single data set with 95% confidence intervals (– –) found using log-likelihoods as described in [34]. The true values of 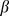 used in the SIR simulation (circles).

Figures 1 and 2 demonstrate that our estimation approach gives a good estimate for 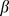 using data from the SIR simulations. In Figure 1, the circles show the true parameter values used for all 10,000 simulations. The solid line shows the mean of the estimated values for the parameters and the dashed lines show the 2.5 and 97.5 quantiles for the parameters estimated from all 10,000 simulations (giving 95% confidence intervals for these estimates). Figure 2 shows the estimates for a single simulated data set randomly selected from the pool of 10,000 simulations. The solid line shows the estimated parameters while the true parameters are shown with circles. The dashed lines show the 95% confidence intervals. The true parameter values are included inside these confidence intervals.

**Figure 3:**
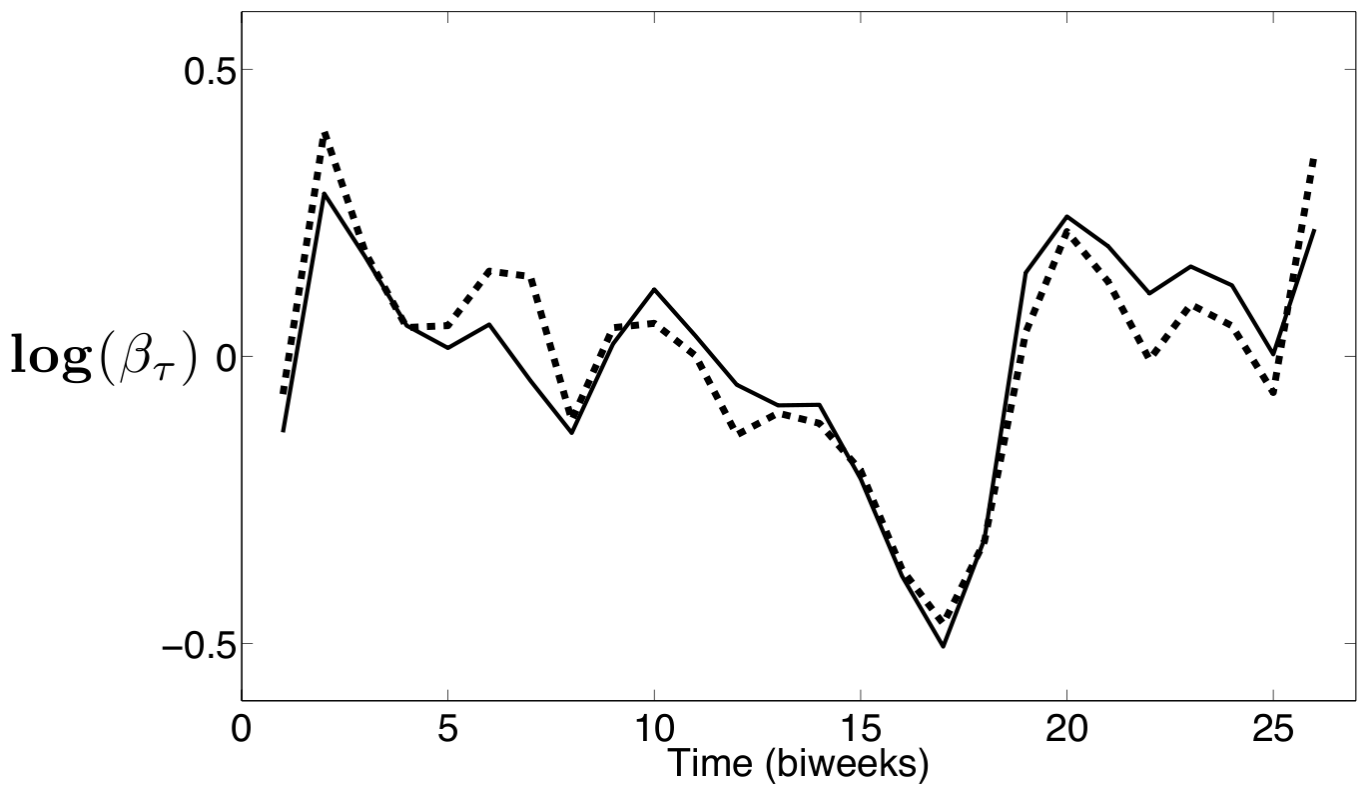
A comparison of seasonal transmission parameters estimated for London. The values estimated in this work (– –) are overlaid with those reported by Finkenstädt and Grenfell [11] (—).

To further validate this approach, we then performed estimations with the London data that had been used in other studies and found our results to be consistent with other literature estimates [2], [12], [14], [16]. Our estimate of seasonality in 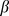 for London is similar to that obtained by Finkenstädt and Grenfell [11] for England and Wales over the same time period. Figure 3 shows a comparison of our estimated 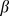 with that estimated by Finkenstädt and Grenfell [11]. The seasonality observed shows a small drop in the transmission parameter at the Easter break (biweek 8), and a large drop at the summer break (biweeks 15–18). This observation is in agreement with the proposal that transmission of measles is correlated with school holidays.

Our estimates are very similar to others in the literature, and our solution approach is also very fast. The run time for the London estimation is under 5 seconds even though this estimation included 21 years of data and estimated the susceptible population and reporting fraction simultaneously with the model parameters. The computational time required for all estimates reported in this work using real case data is given in Table 1. Given our ability to accurately estimate known parameters given simulated data, and the similarity between our estimates and other estimates from the literature using London data, we are confident that our estimation procedure offers a fast, reliable method to estimate seasonal parameters in infectious disease models.

**Table 1:** Problem size and solution times for the London, New York City (NYC), and Bangkok estimation problems studied in this paper.

City	Model	Variables	Constraints	CPU Time (sec)^a^
London	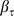	3878	3847	4.5
	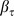	3696	3665	2.3
NYC	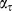	3671	3640	3.2
	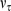	3671	3640	3.2
	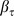	2240	2183	0.6
Bangkok	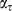	2215	2158	5.6
	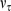	2215	2158	0.6

a All problems were solved on a 2.13 GHz Intel Core 2 Duo processor and times are reported in seconds.

### Estimation Results for Seasonality in Different Model Parameters

In this section, we show the estimation results for the three different problem formulations SVTP, SVEP, and SVIS, addressing seasonality in the transmission parameter, the exponential parameter, and the introduction of susceptibles respectively. Each of these three formulations is solved using measles data from both New York City and Bangkok, two locations with significantly different school holiday schedules.

**Figure 4:**
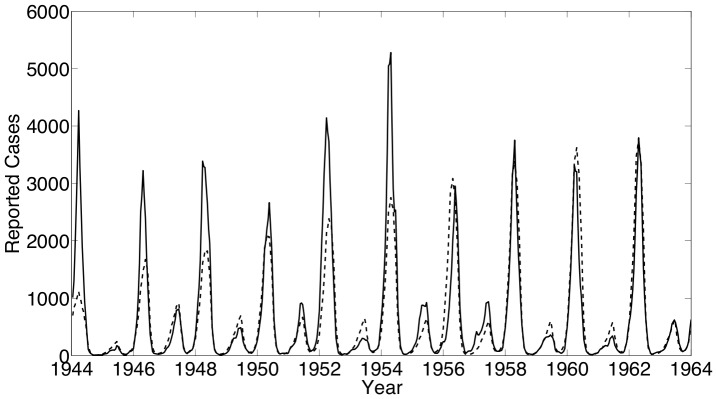
New York City results: The estimated number of reported cases (– –) with the actual number of reported cases (—) of measles.

**Figure 5:**
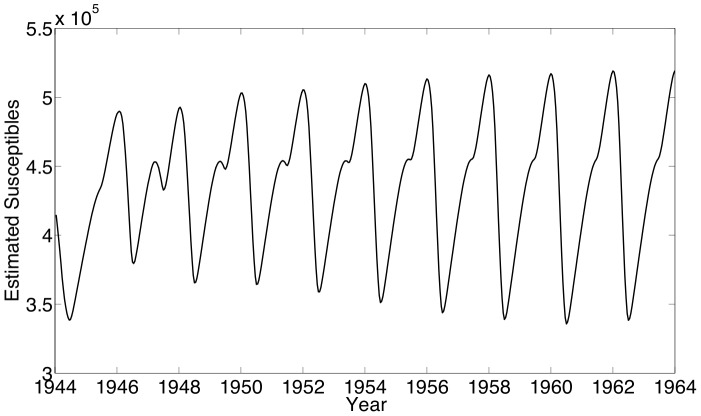
New York City results: The estimated number of individuals susceptible to measles.

**Figure 6:**
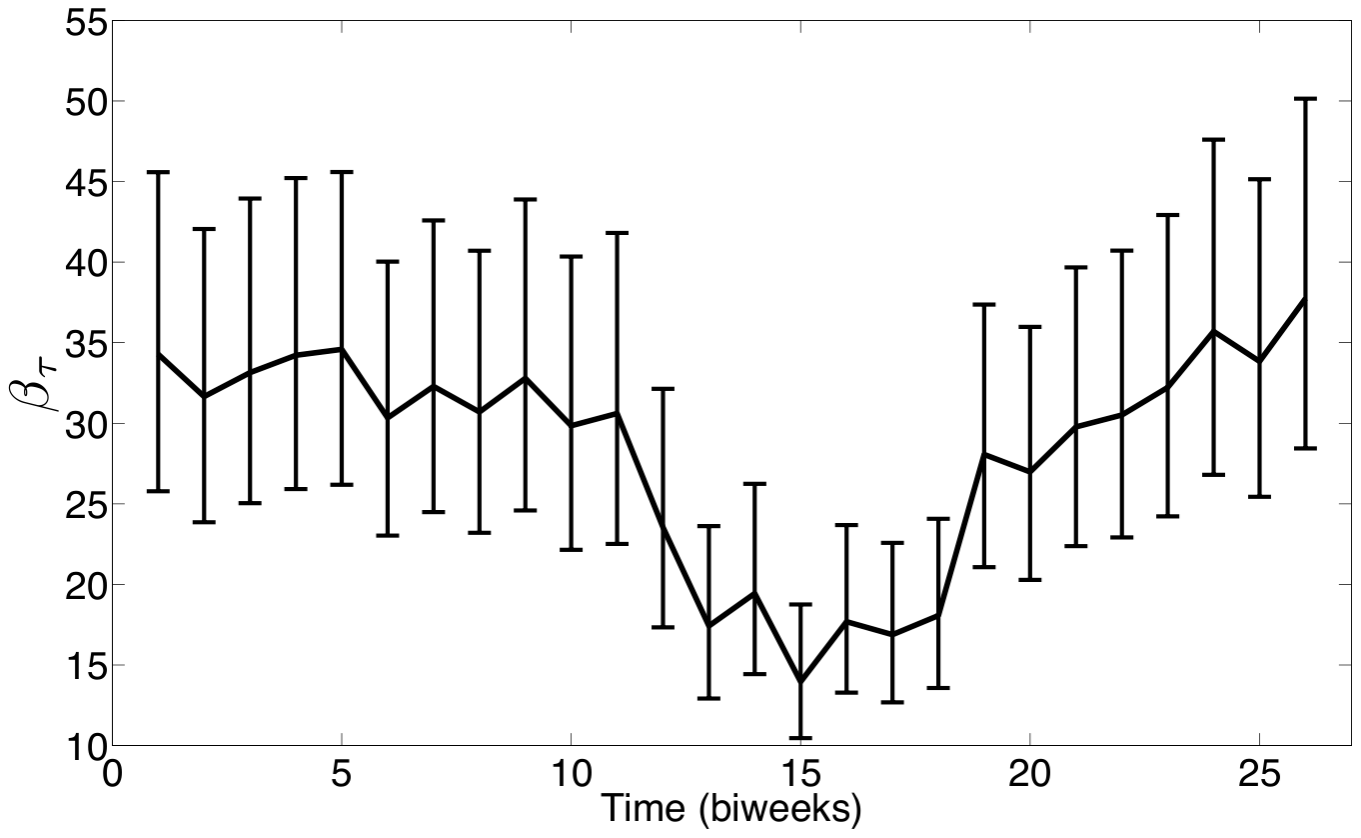
Estimated 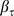 for measles in New York City with 95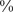 confidence intervals (—).

The first set of estimation results are shown for formulation SVTP using measles data from New York City. Our estimates using this data yield an estimated number of reported measles incidence that fits reasonably with the actual reported incidence data as shown in Figure 4. The mismatch shown at the beginning of the time horizon may be due to our assumption that the reporting fraction varies linearly in time. The biennial periodicity seen in the case counts and the estimated number of susceptibles (Figure 5) is consistent with expectations for endemic measles in cities with a low birth rate. Our estimate of 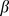 using NYC measles data is shown in Figure 6, and the observed seasonality coincides strongly with the school term summer break which occurred over biweeks 11–17. While the confidence intervals shown in Figure 6 seem large, recall that we determine these intervals by fixing the value of a single parameter and optimizing over the remaining parameters (i.e., re-estimating the remaining parameters) to generate profile likelihoods.

**Figure 7:**
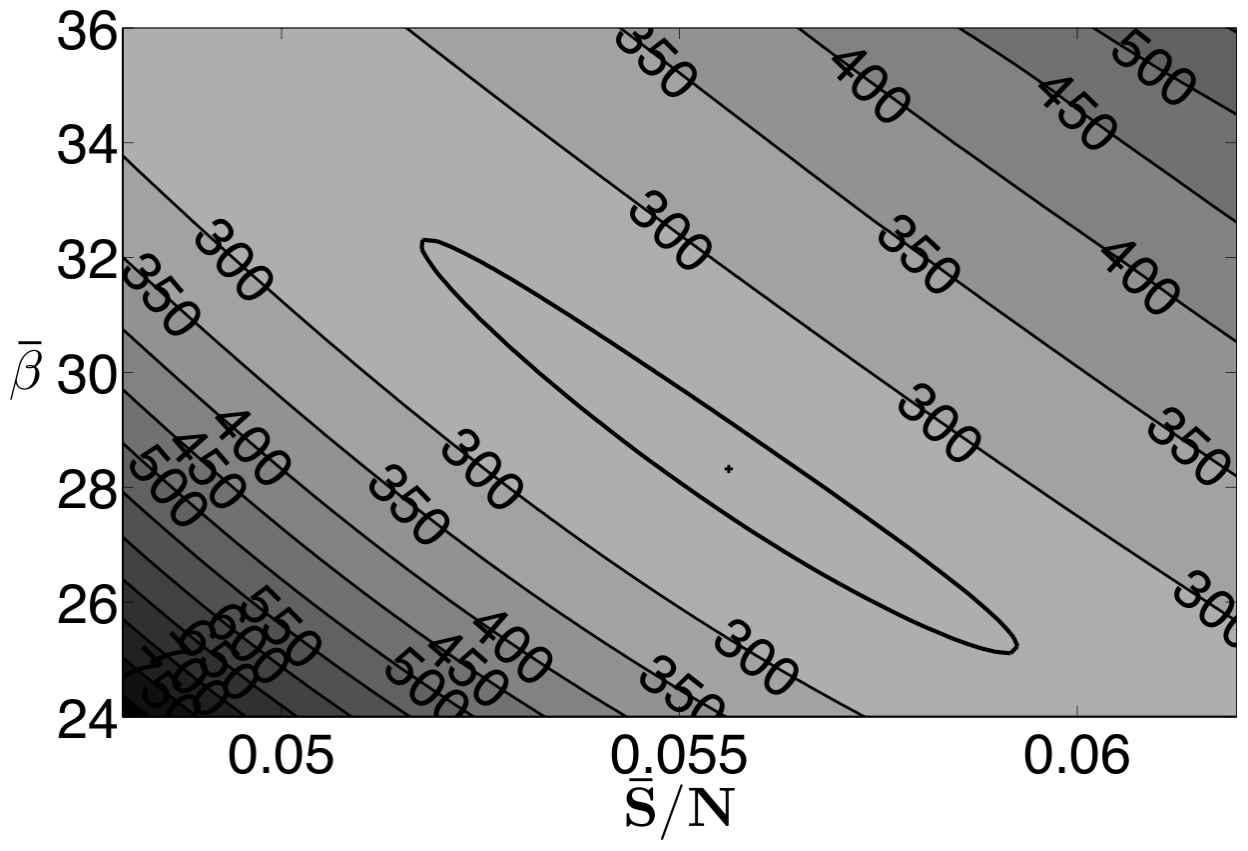
New York City results: 95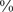 confidence region for 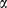 and 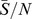.

**Figure 8:**
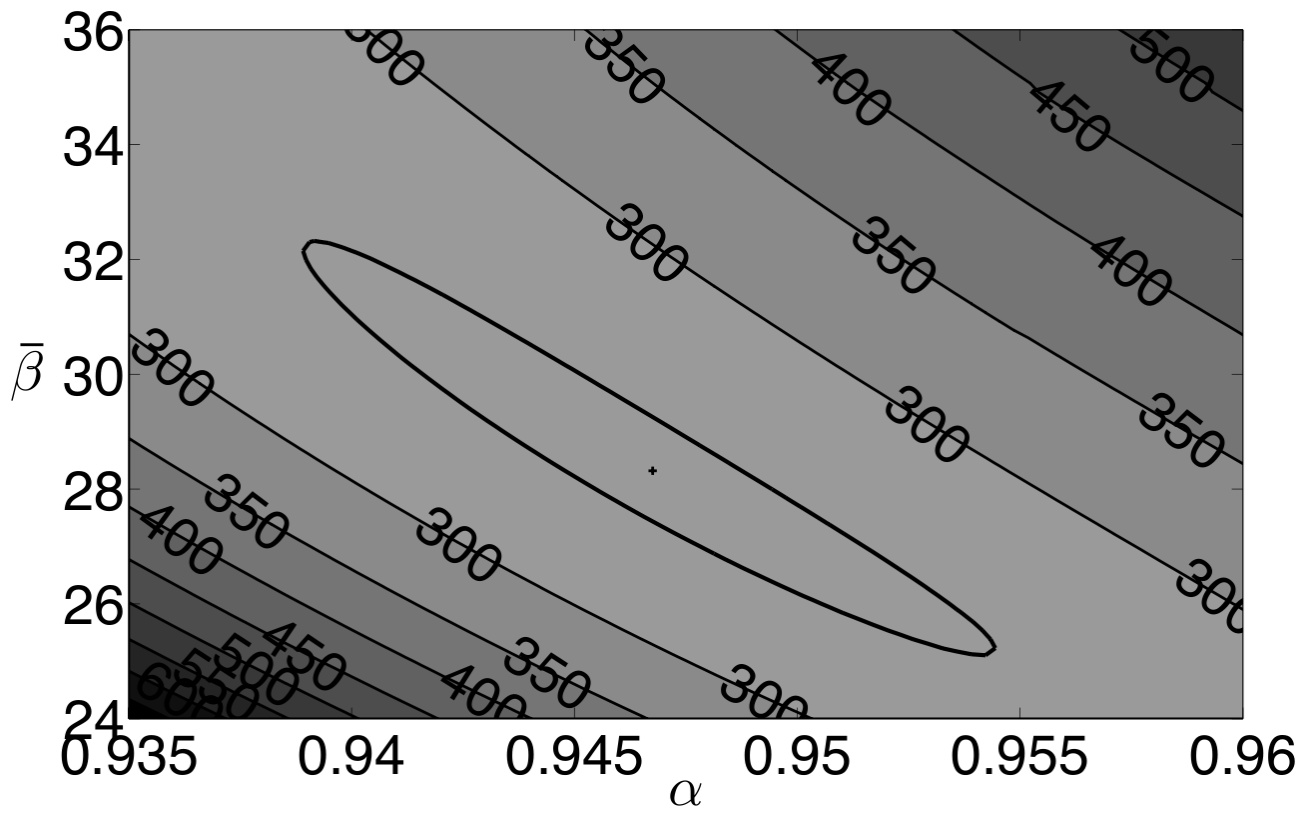
New York City results: 95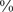 confidence region for 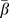 and 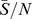.

**Figure 9:**
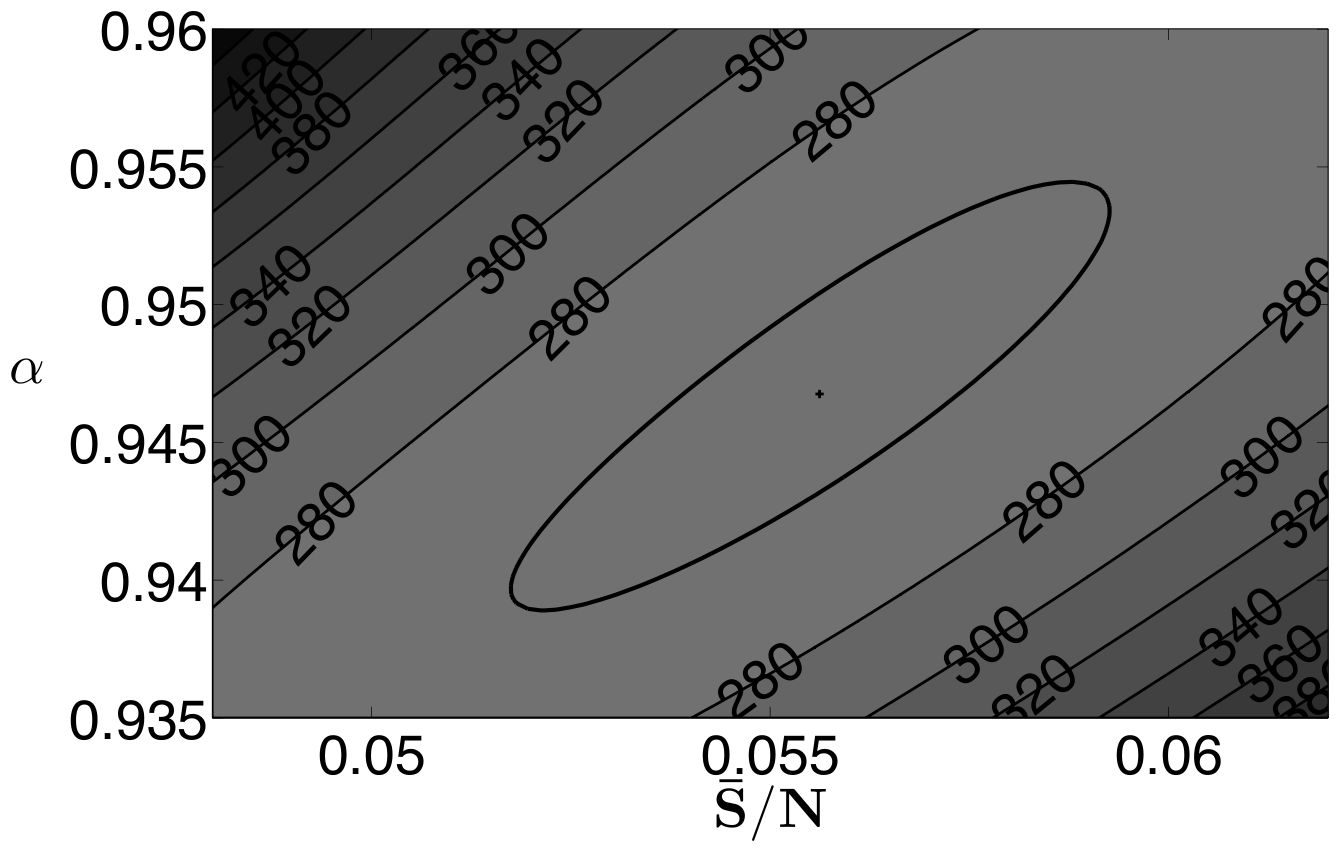
New York City results: 95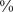 confidence region for 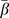 and 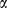.

At the solution of the estimation problem, the optimization package Ipopt indicates that the reduced-Hessian is positive definite, which implies that the estimated parameters are locally unique. However, in addition to single variable confidence intervals, we are also interested in confidence regions where parameter values can be expected. Using the procedure outlined in Rooney and Beigler [34], we construct pairwise confidence regions for the mean of the transmission parameter, the mean of the susceptible population, and the exponential parameter 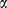, based on the likelihood ratio test. Figure 7 shows the relationship between the exponential parameter (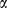) and the mean susceptible fraction (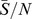), Figure 8 shows the relationship between the mean of the transmission parameters (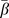) and the mean susceptible fraction (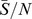), and Figure 9 shows the relationship between the mean of the transmission parameters (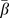) and the exponential parameter (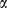). In all figures the plus sign indicates the optimal solution, and the bold line indicates the extent of the 95% confidence region. All three regions have similar shapes and show some correlation between the parameters. High values of 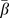 correspond to lower values of 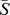 and 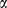. This is reasonable since an increase in the infection term 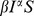 caused by a higher 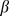 could be offset somewhat by a reduction in either 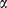 or 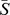. An increase in 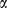 corresponds to a higher value of 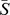 which seems contrary to the previous results, however, when constructing these intervals, we optimize over the remaining variables and a lower value of 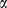 corresponds to a higher value of 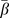. Furthermore, the relative range of 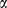 in the confidence region is much smaller than that of 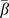 and 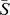. While not shown here for brevity, confidence regions with similar characteristics were found for the other data sets used in this paper.

**Figure 10:**
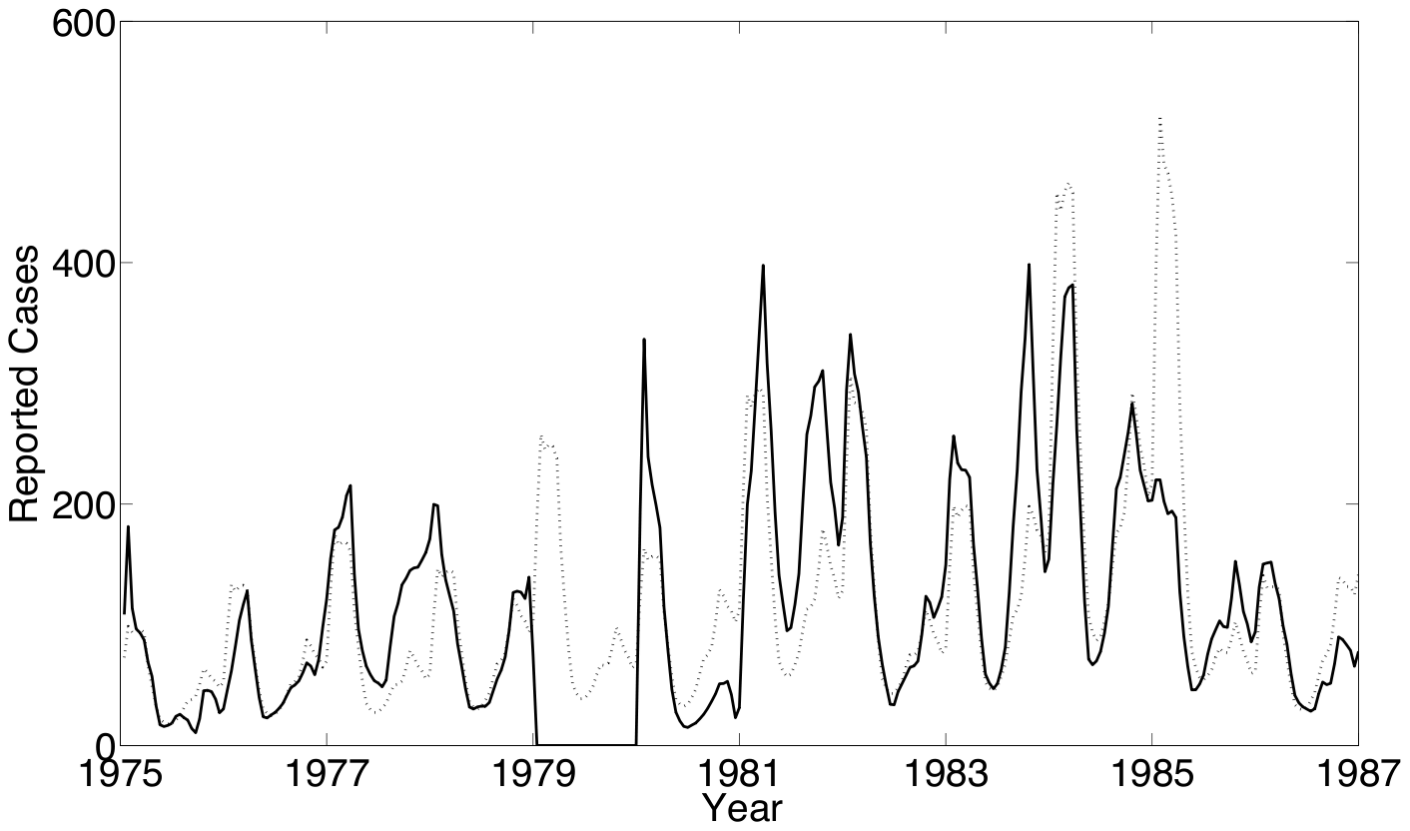
Bangkok results: The estimated number of reported cases (– –) with the actual number of reported cases (—) of measles. Note: Case data is unavailable for 1979.

The Bangkok data allows us to perform estimates for a location with a very different social environment and school schedule than NYC. These case counts suffered from a much lower reporting fraction and even missing data during one year (1979). Still, the estimated reported measles incidence gives a remarkably good fit to the actual reported incidence data as shown in Figure 10.

**Figure 11:**
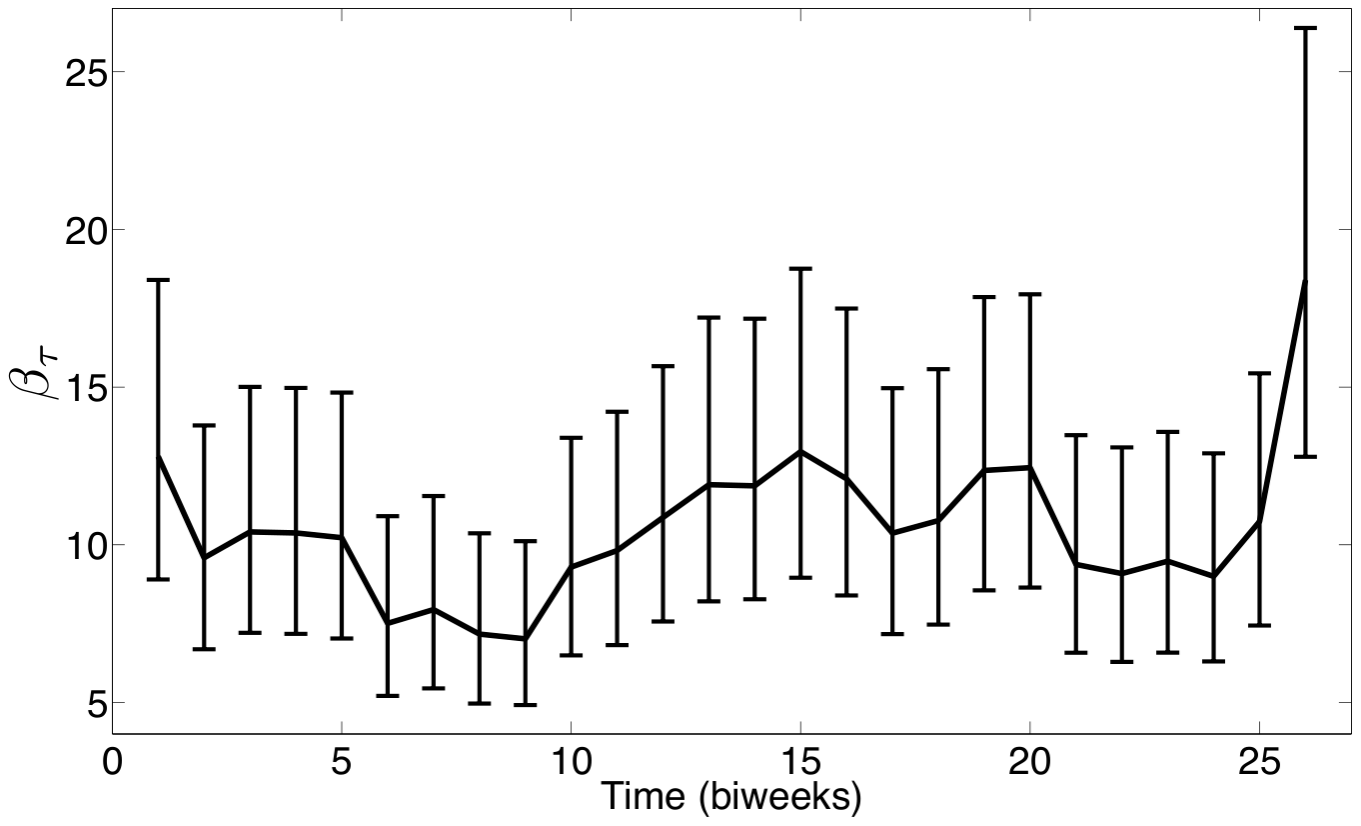
Estimated 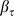 for measles in Bangkok with 95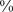 confidence intervals (–).

Our estimate of 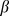 for Bangkok is shown in Figure 11, and, as expected, the estimated transmission profile is very different from those estimated for both London and NYC. However, the observed seasonality again appears to be correlated with the school term holidays that occur from the beginning of March through the middle of May and the entirety of October (corresponding approximately to biweeks 5–9 and 20–21 respectively). This estimated seasonality does not appear to be as strong as that seen in the NYC estimate, but this could be due to the high degree of under-reporting in this data set. Our estimates show that only about 1% of cases are reported at the beginning of the time horizon and that this fraction increases to only about 5% of the cases being reported by the end of the time horizon. This low reporting fraction allows for significant noise to be present in the available data which could reduce our ability to estimate seasonality. The complete set of parameter estimation results and confidence intervals for the SVTP estimates using NYC and Bangkok data are given in Table 2.

**Table 2:** Estimated parameters with 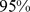 confidence intervals for measles in New York City and Bangkok using a seasonal transmission parameter.

	NYC Measles			Bangkok Measles		
	Est	Low	High	Est	Low	High
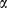	0.9468	0.9405	0.9530	1.0137	1.0056	1.1062
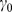	0.07363	0.06599	0.08188	0.01187	0.01013	0.01379
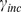	9.57E-5	6.30E-5	1.29E-4	1.00E-4	8.30E-5	1.17E-4
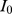	11360	8750	14810	6810	5120	9080
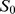	414800	393600	435900	352200	339500	362400
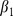	34.28	25.74	45.60	12.80	8.86	18.40
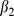	31.66	23.79	42.07	9.58	6.60	13.82
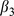	33.14	24.95	43.94	10.41	7.16	15.05
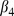	34.22	25.83	45.25	10.37	7.11	15.04
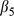	34.59	26.17	45.63	10.22	6.99	14.86
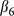	30.33	22.94	40.05	7.51	5.14	10.94
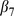	32.29	24.41	42.68	7.94	5.44	11.55
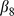	30.70	23.11	40.77	7.16	4.92	10.40
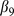	32.79	24.52	43.89	7.01	4.83	10.17
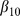	29.85	22.12	40.38	9.29	6.40	13.47
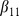	30.61	22.48	41.88	9.82	6.77	14.23
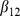	23.54	17.32	32.18	10.86	7.49	15.74
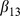	17.42	12.90	23.62	11.91	8.21	17.26
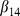	19.44	14.42	26.26	11.87	8.18	17.20
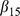	13.96	10.40	18.77	12.95	8.93	18.78
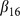	17.69	13.21	23.71	12.09	8.34	17.53
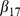	16.88	12.62	22.59	10.37	7.16	15.01
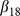	18.07	13.53	24.14	10.77	7.44	15.58
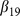	28.07	21.04	37.46	12.36	8.54	17.88
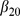	26.98	20.23	36.01	12.44	8.60	17.98
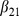	29.77	22.32	39.72	9.37	6.49	13.52
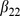	30.52	22.88	40.72	9.09	6.29	13.11
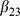	32.22	24.15	42.99	9.48	6.56	13.67
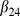	35.70	26.76	47.63	9.00	6.22	12.99
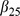	33.84	25.36	45.14	10.74	7.42	15.50
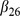	37.74	28.37	50.17	18.39	12.75	26.43

In an effort to investigate seasonality in other model parameters, the SVEP model formulation is used to estimate seasonal exponential parameters 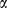 using data from both New York City and Bangkok. This formulation includes a time-invariant transmission parameter and seasonal exponential parameters 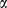 (5). Similar to the estimated profiles using SVTP, these estimations show a seasonal profile for 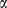 that appears strongly correlated with the school holiday schedule.

**Figure 12:**
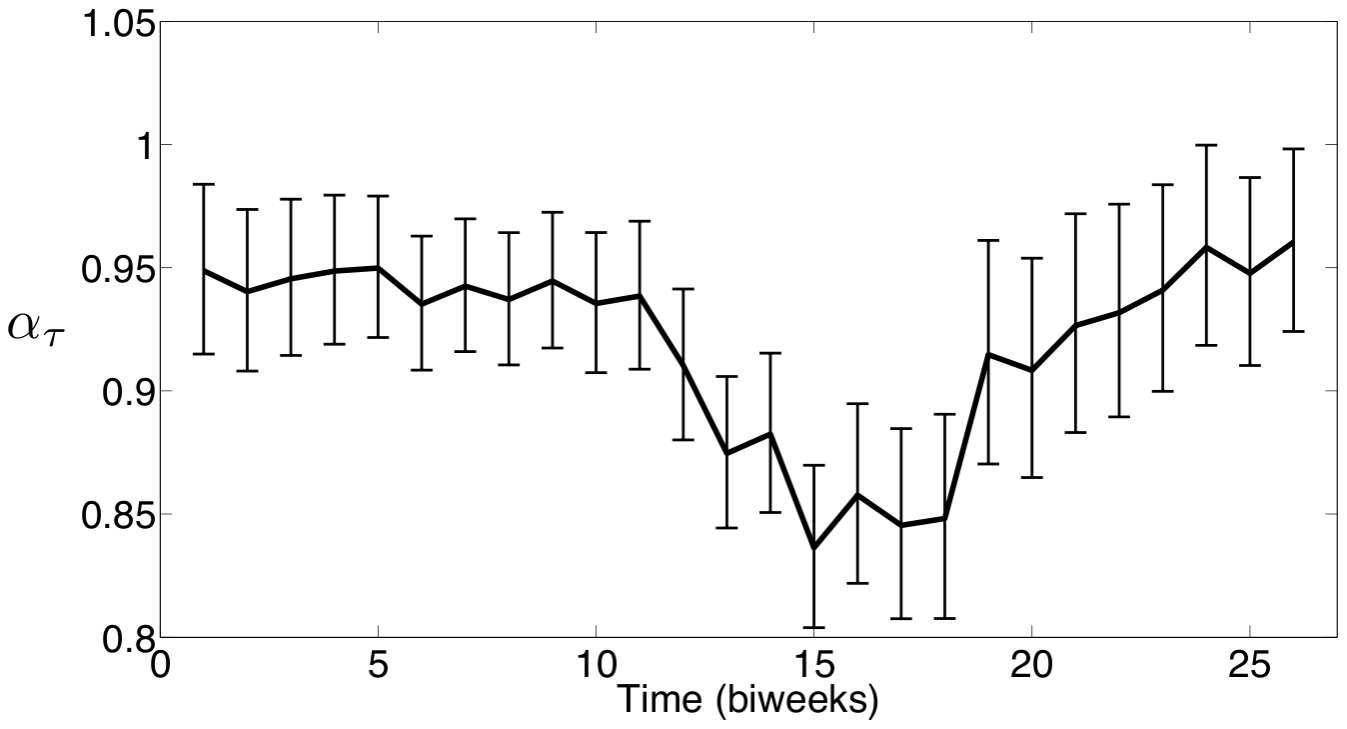
New York City estimates of seasonal exponential parameter 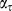 with 95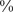 confidence intervals.

**Figure 13:**
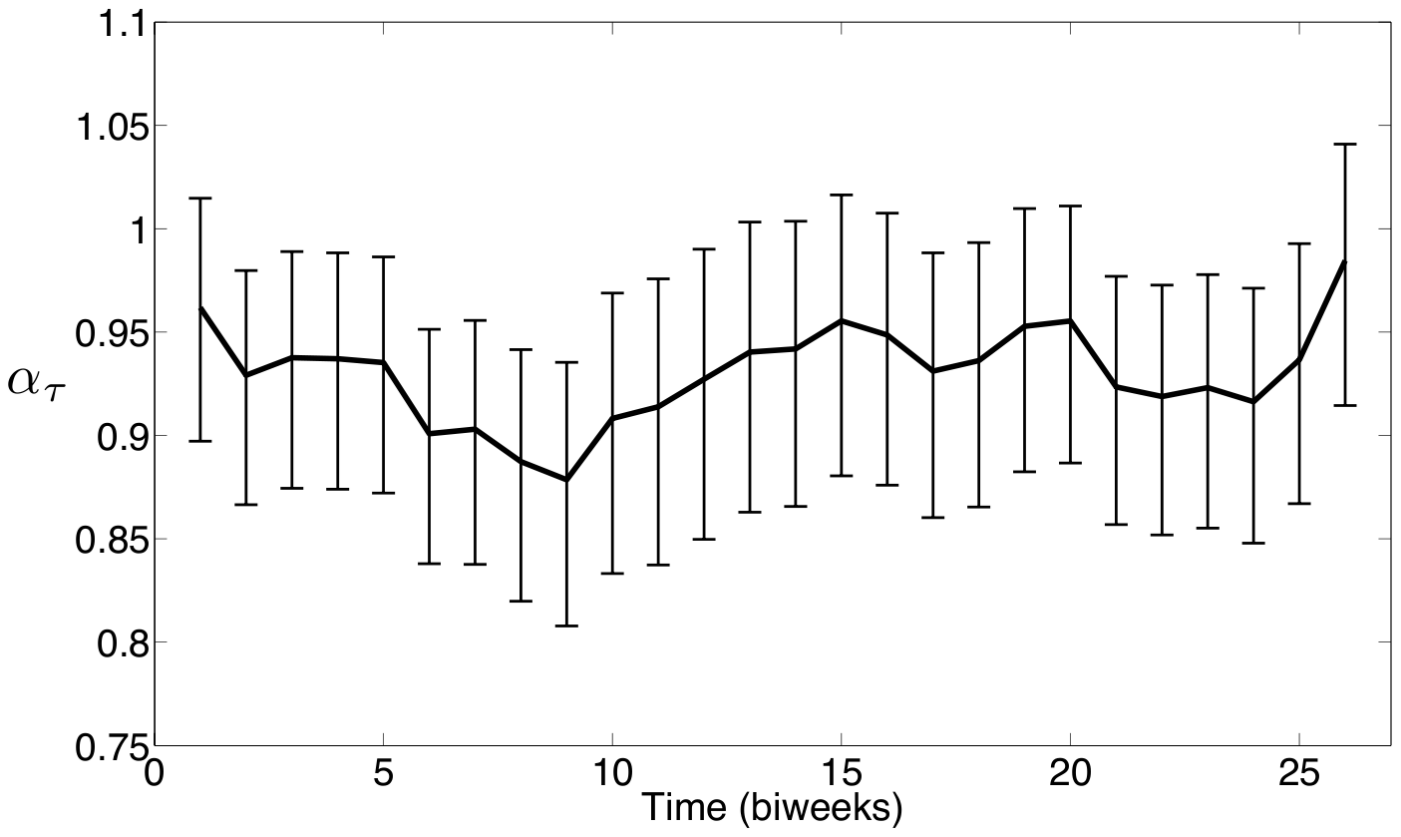
Bangkok estimates of seasonal exponential parameter 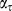 with 95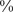 confidence intervals.

The seasonal 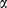 estimated using the New York City measles data is shown in Figure 12, and the seasonal 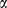 estimated using the Bangkok measles data is shown in Figure 13. After scaling, the estimated seasonality in 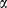 is almost identical to the profiles estimated for the seasonal 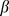
's. This demonstrates that while seasonality provides a mechanism to capture the dynamics seen in the data, the actual implementation of the seasonality into the model can vary. This also shows that 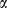 and 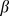 could be describing some combination of several physical phenomena, and care must be taken when interpreting the underlying cause of the seasonality.

**Figure 14:**
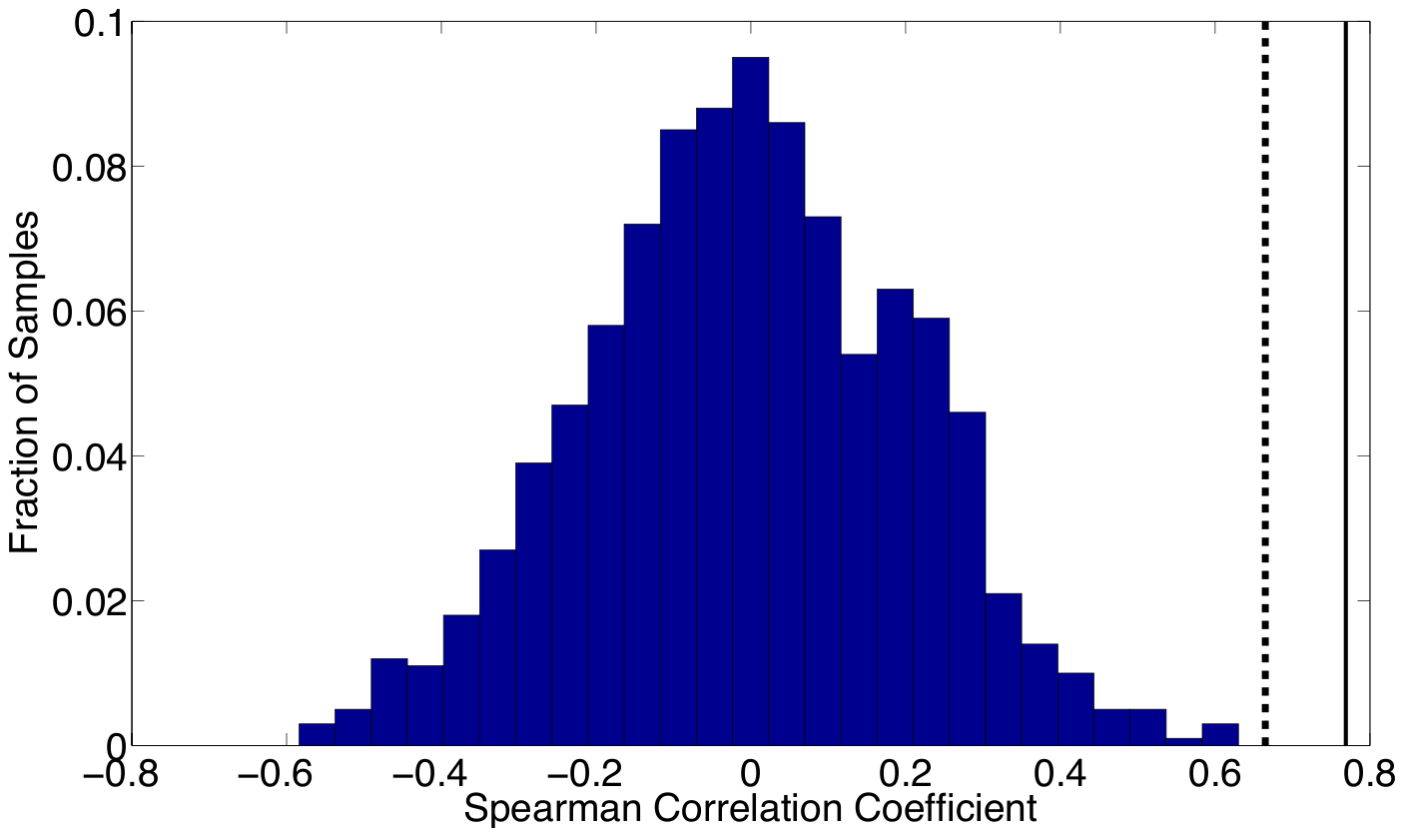
Spearman correlation coefficients computed for New York City are shown. The coefficient calculated using reported holiday schedules and estimated parameters are shown by the dashed line. The coefficient computed using a holiday schedule shifted forward by one biweek is shown by the solid line. The histogram shows the distribution of correlations that were computed between the reported holiday schedule and 1,000 randomly ordered vectors of our parameter estimates.

**Figure 15:**
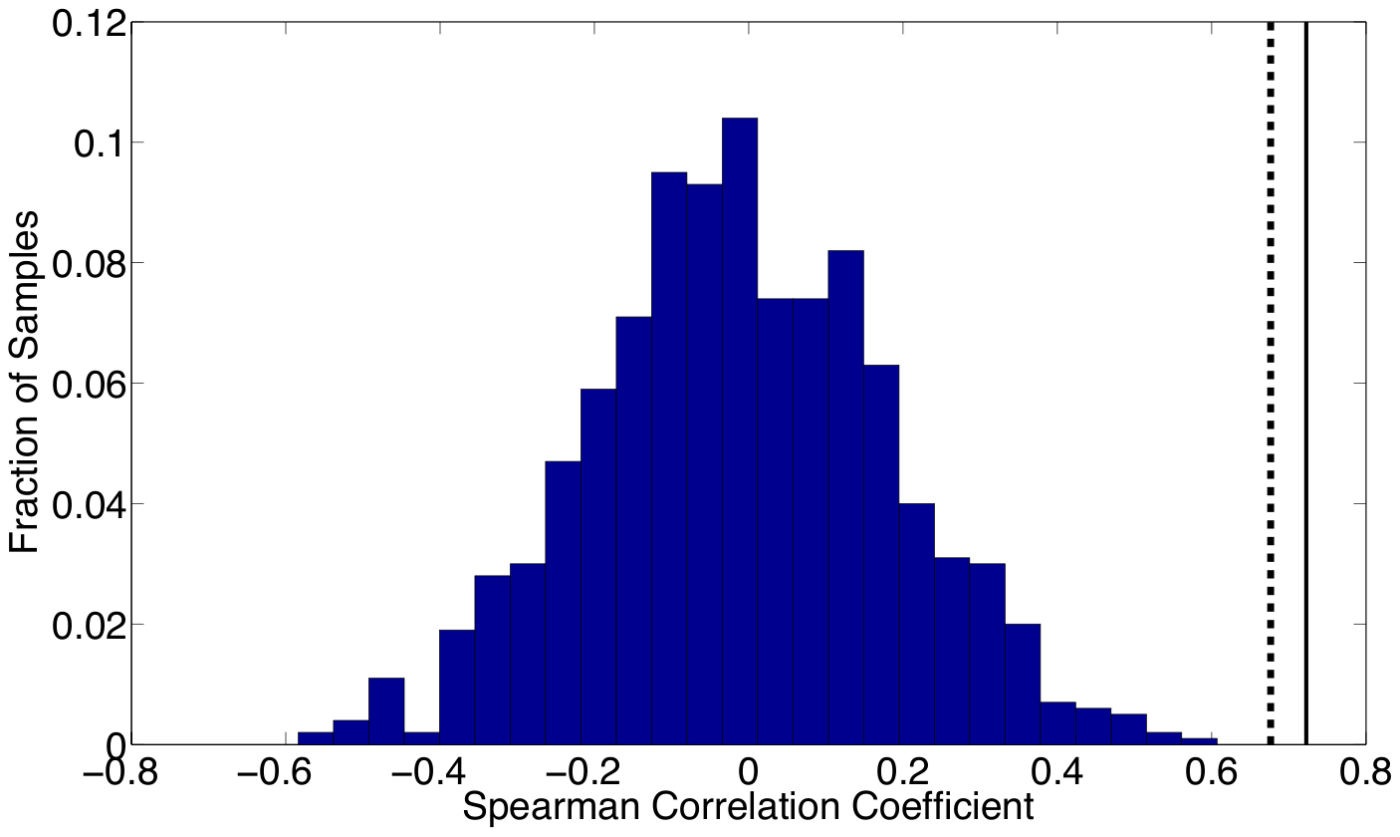
Spearman correlation coefficients computed for Bangkok are shown. The coefficient calculated using reported holiday schedules and estimated parameters are shown by the dashed line. The coefficient computed using a holiday schedule shifted forward by one biweek is shown by the solid line. The histogram shows the distribution of correlations that were computed between the reported holiday schedule and 1,000 randomly ordered vectors of our parameter estimates.

To compare these results numerically, Spearman correlation coefficients are computed to compare our estimated seasonality with school holidays. For the school term profile we construct a 0/1 sequence where a 1 indicates that school is in session for a particular biweek and a 0 indicates that school is on holiday. Figure 14 shows the Spearman correlation coefficient compute using reported holiday schedules and the estimated seasonal 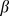 parameters for New York City. We also computed the Spearman correlation coefficient using a holiday schedule that is shifted forward by one biweek, and this coefficient is even higher. This shift is not unreasonable given that the data was reported monthly but was resampled into a biweekly form suitable for our formulation. The histogram in this figure shows the distribution of correlations that were computed between the reported holiday schedule and 1,000 randomly ordered vectors from our estimated 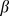 values. This histogram demonstrates that the correlation between school holidays and random seasonality in the parameters is normally distributed about zero, while the correlation between the holidays and our estimated seasonality is very strong (above the 95% confidence level). Figure 15 displays the same calculations as Figure 14 except using Bangkok school holidays and parameter estimates.

The same calculations are performed using the estimated seasonal 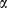 values with almost identical results. The Spearman correlation coefficients for New York City and Bangkok using the unshifted holidays and seasonal 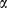
's are 0.62 and 0.61 respectively. Using shifted holidays, these correlation coefficients are 0.76 and 0.72 for New York City and Bangkok respectively. These results strongly support our belief that our estimated seasonality is correlated with school holidays. Furthermore, the estimated seasonality in 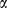 and 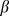 are strongly correlated with each other. The correlation coefficient between the estimated seasonal 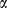 and seasonal 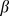 for New York City is 0.99, and the correlation coefficient between the estimated seasonal 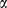 and seasonal 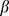 for Bangkok is 0.97.

For the time periods under consideration, we have only yearly birth data. For the previous estimation results (SVTP and SVEP) we assumed that the birth rates were constant throughout the year. Formulation SVIS estimates seasonality in the introduction of susceptibles into the 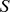 compartment. The SVIS model formulation is used for estimates using data from New York City and Bangkok. This formulation contains time-invariant transmission and exponential parameters and considers seasonality in births by including a weighting factor 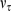 that must be estimated.

**Figure 16:**
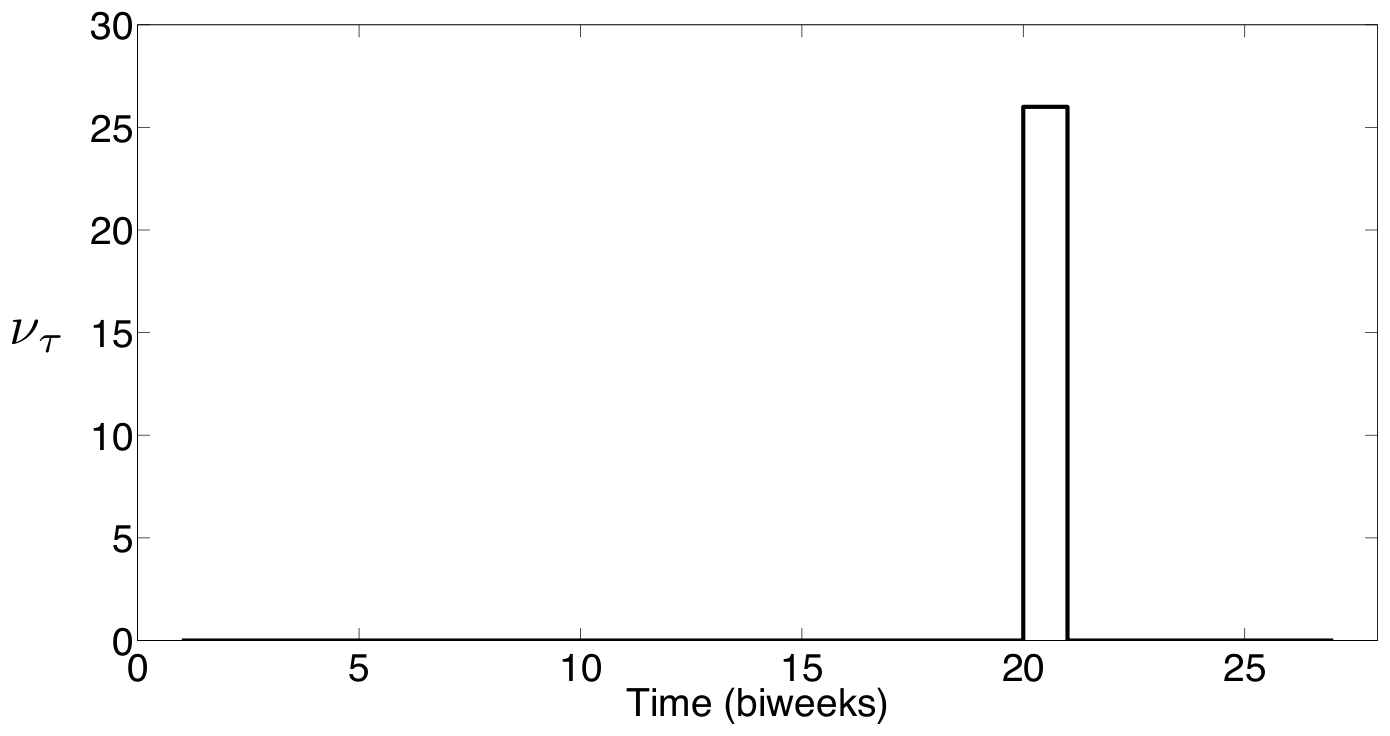
New York City estimates of seasonal weight on births, 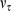.

**Figure 17:**
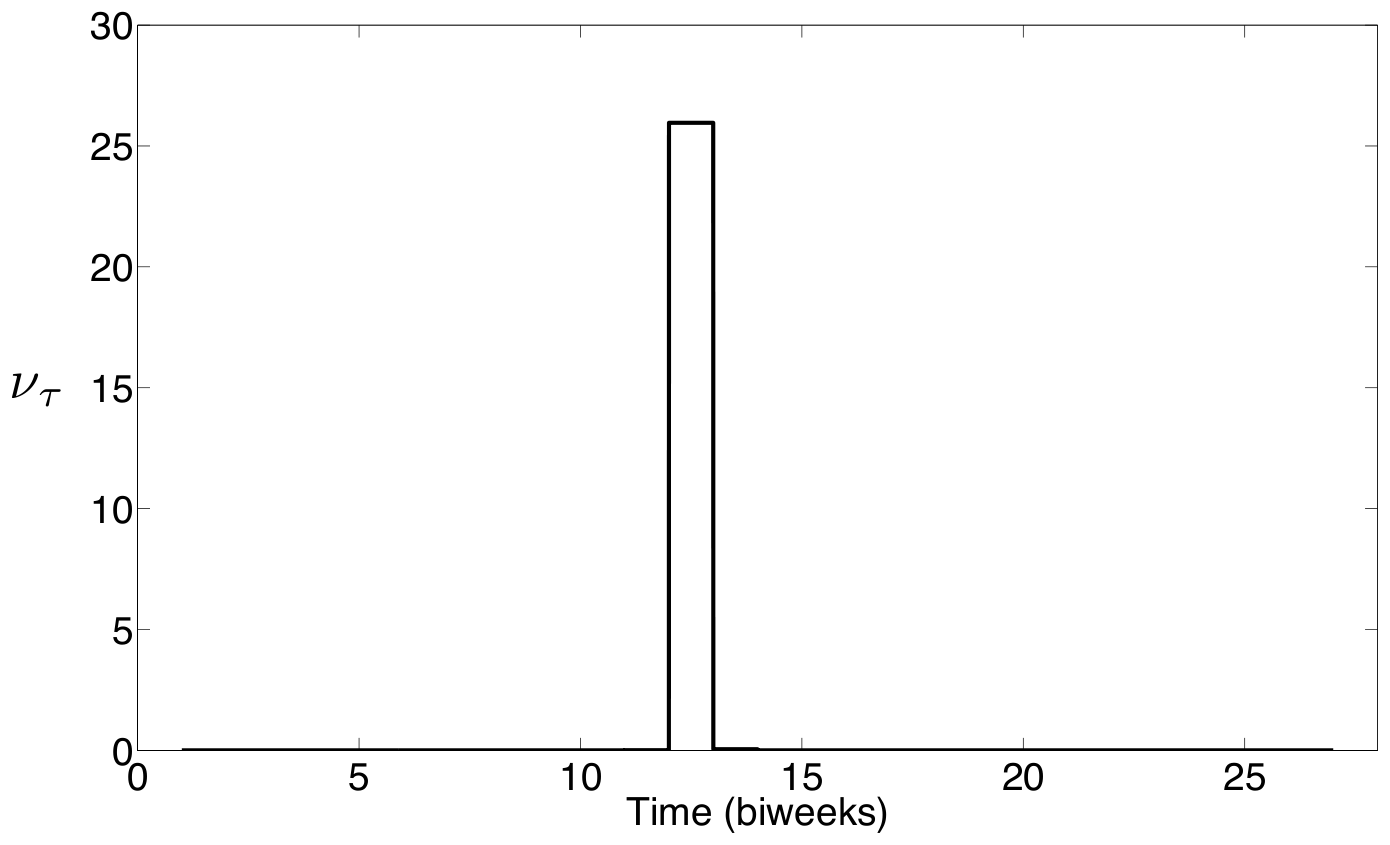
Bangkok estimates of seasonal weight on births, 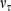.

The seasonal profile for 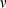 estimated using the New York City measles data is shown in Figure 16, and the seasonal profile for 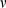 estimated using the Bangkok measles data is shown in Figure 17. Just as with previous estimates, these results show strong correlation between the seasonality observed in measles case data and school term holidays. For New York City, 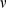 is essentially zero except at the end of September which is immediately following the start of the fall semester of school. For Bangkok, 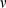 is essentially zero except at the beginning of June. These results do not show a seasonal variation that is consistent with estimated seasonal profiles for the other two parameters, however, these profiles are very interesting in that they are still highly correlated with the school schedules in both settings. The seasonal profiles providing an optimal fit to the data show complete introduction of the new susceptible immediately following the major holiday at the start of the school year. This is consistent with the idea that susceptible children impact the observed measles dynamics when they enter the school population.

## Conclusions

The development of inference tools for infectious disease models remains an important challenge to better understand disease dynamics and develop more effective control strategies. This work has demonstrated the flexibility inherent in large-scale NLP techniques and the ability of these techniques to efficiently estimate transmission parameters in multiple disease models using measles case count data. We demonstrated this efficiency and flexibility using three model formulations and four data sets. In all cases, including for time-series data sets of over 20 years, we were able to perform the estimations in less than 6 seconds. This computational efficiency and flexibility opens the door for investigating many alternative model formulations and encourages use of these techniques for estimation of larger, more complex time-discretized models like those with age-dependent dynamics, more complex compartment models, and spatially distributed data.

We validated our estimation approach by performing 10,000 estimations using simulated case data, and to demonstrate the flexibility of our approach, we presented estimations using measles case data from 3 different models. The first model we presented used a seasonally varying transmission parameter 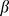. We first validated our approach by estimating seasonal transmission parameters using both simulated data and real measles data from London. We performed 10,000 estimations on simulated data (with different noise realizations) and showed that the approach was able to effectively recover the true seasonal transmission parameter. Furthermore, this approach estimates seasonal transmission parameters for London that are consistent with other estimates in the literature [23], [35].

Using real measles case data from both New York City and Bangkok, we estimated using 3 formulations, SVTP, SVEP, and SVIS, considering seasonality in the transmission parameter, the exponential parameter, and the introduction of new susceptibles. In all cases, the estimated seasonality showed correlation with school schedules. This is especially important given that the school schedules differ significantly for these two locations. The profile estimated using seasonal 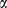
's was practically identical to that estimated using seasonal 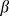
's. This result might not be too surprising, but this does highlight that care must be taken when relating the estimated seasonality to particular system phenomena (e.g., contact rate).

Perhaps more interesting are the estimation results for the model with a seasonal weighting of the births. Here, instead of assuming that new susceptibles always entered the population uniformly throughout the year, the model was formulated so that susceptibles could enter the population in any seasonal pattern. These estimation results show that all new susceptibles were introduced to the population immediately following long school holidays to best capture the dynamics observed in reported measles cases. Since all births clearly do not actually occur at this time, this result is consistent with the idea that the susceptible children impact observed measles dynamics when they enter the school population.

### Future Work

The flexibility and efficiency of our solution approach will allow us to tackle more difficult problems. Along with disease case counts, some data sets also include age of infection information. This additional information could be used in a model with seasonal and age dependent transmission. A model of this type would not only give an age of infection distribution more consistent with the actual data, but could also allow the incorporation of the age of infection data in a natural way.

Additionally, while measles can be endemic in larger cities, it is seen to die out in smaller communities with re-emergence arising from infections imported from surrounding metropolitan areas. Spatially distributed models have been used to estimate the impact of disease transmission from large cities to surrounding areas, but these problems can be very large, making it impractical to also estimate seasonality in model parameters. The power of large-scale NLP solvers could allow for simultaneous estimation of seasonality and spatio-temporal effects over large spatially distributed data sets.
